# Morphological evidence for phages in *Xylella fastidiosa*

**DOI:** 10.1186/1743-422X-5-75

**Published:** 2008-06-06

**Authors:** Jianchi Chen, Edwin L Civerolo

**Affiliations:** 1 San Joaquin Valley Agricultural Sciences Center, Agricultural Research Services, United States Department of Agriculture, Parlier, California, 93648, USA

## Abstract

Presumptive phage particles associated with *Xylella fastidiosa *strain Temecula-1 grown in PW broth were observed by transmission electron microscopy (TEM) in ultrathin sections of bacterial cell-containing low speed centrifugation pellets and in partially purified preparations from CsCl equilibrium centrifugation density gradients.  Ultrathin-sectioned cell pellets contained icosahedral particles of about 45 nm in diameter. Samples collected from CsCl density gradients revealed mostly non-tailed icosahedral but also tailed particles. The icosahedral particles could be divided into two types: a large type (about 45 nm) and a small type (about 30 nm). Filamentous phage-like particles (17 × 120 to 6,300 nm) were also observed. The presence of different types of phage-like particles resembling to those in several bacteriophage families provides new physical evidence, in addition to *X. fastidiosa *genomic information, that *X. fastidiosa *possesses active phages. This is the first report of phage particles released in *X. fastidiosa *cultures.

## Findings

*Xylella fastidiosa *[1] is a Gram negative plant pathogen causing many economically important diseases including Pierce's disease (PD) of grapevine, almond leaf scorch disease and citrus variegated chlorosis disease. Because of nutritional fastidiousness, many biological aspects of the bacterium including the occurrence of phages are difficult to study. Analyses of whole genome sequences of *X. fastidosa *strains identified many prophage sequences [2-5], including putative *Siphoviridae *[2,4], *Podoviridae *[6] and *Inoviridae *[3] phages. Yet, physical evidence for the presence of phage particles in *X. fastidiosa *is very limited. Lauzon and Miller [7] reported the association of particles resembling phages in the families *Microviridae *and *Podoviridae *with *X. fastidiosa*. However, only limited details regarding the origin(s) or nature of these particles were provided. Chen et al. [6] reported a phage DNA sequence of 547 bp from the genome of a PD strain isolated in Florida. The sequence shared high similarity to that of an integrase gene in the *Podoviridae *phage family. Interestingly, this sequence is absent in the whole genome sequence of a California PD strain Temecula-1, but is present in other California PD strains. In this paper, we report our observations of presumptive phage particles in a *X. fastidiosa *PD strain through transmission electron microscopy (TEM).

Phage observations were first made with intact bacterial cells. *X. fastidosa *strain Temecula-1 was cultured in 30 ml of PW broth medium [8] for 30 days at 28 C. Before bacterial cell collection, a loop of bacterial culture was streaked on PW plate and incubated at 28 C to check for possible contamination based on culture characteristics (slow growing opalescent colonies with entire smooth margin) as well as PCR [9]. Bacterial cells were then collected by centrifugation at 3,000 g for 30 minutes. Cell pellets were suspended in 1 ml of TE (10 mM Tris-HCl, pH 8.0 and 50 mM EDTA) buffer, transferred to a 1.5 ml microfuge tube and collected by centrifugation at 3,000 g for 20 minutes. Pelleted cells were re-suspended in 2% glutaraldehyde in 0.1 M sodium cacodylate buffer (pH 7.4). Following rinsing in cacocylate (pH 7.4) buffer, the cells were post-fixed in 1% osmium tetroxide in 0.1 M sodium cacodylate buffer; dehydrated successively in 50%, 70%, 80%, 95%, 100% ethanol and 100% acetone; and embedded in Spurr's embedding medium [10]. For the final step in embedding, cells suspended in Spurr's were dispensed into Beem capsules (Electron Microscopy Sciences, Hatfield, PA) which were placed in the centrifuge tubes and spun so that pellets were at the tips of the capsules for polymerization. Ultrathin (40–50 nm) sections were made, stained with both uranyl acetate and lead citrate [11] and examined in a FEI Tecnai 12 transmission electron microscope. Images were made with a Megaview III digital camera using analysis software.

**Figure 1:**
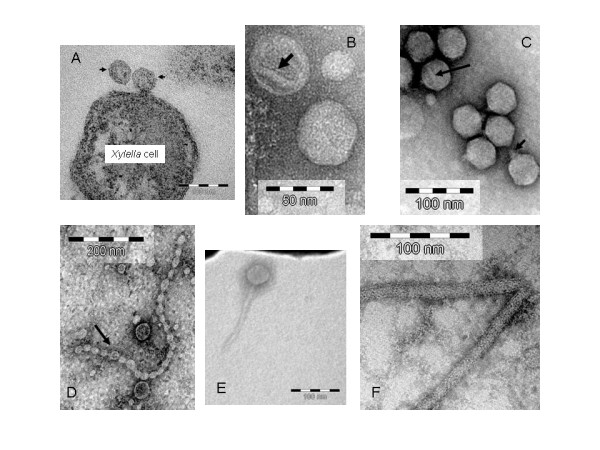
Electron micrographs of: A, Icosahedral phage particles (arrows) associated with a *Xylella fastidosa *cell; B, Icosahedral phage particles showing a "ridge" on the surface (arrow); C, Particles of phage CP2 from *Xanthomonas citri *subsp. *citri*. Long arrow, a surface "ridge" ; Short arrow, a short tail; D, Small type of icosahedral particles in an ordered chain; E, A tailed phage particle. F, Filamentous particles.

As shown in Figure 1A, icosohedral particles were observed outside of and attached to the bacterial cells. Well-defined tails were not apparent, although a faint very short thin structure and resembling a short phage-like tail at a vertex was occasionally observed. The width of these particles was 45.2 ± 8.5 nm (n = 70). The isometric morphology and the size of these particles suggested that these particles were putative virions of bacteriohages, probably in the family *Podoviridae *[12]. Interestingly, TEM images of *X. fastidosa *bacteria published earlier [13] include morphologically similar phage-like particles; however, there was no discussion or interpretation of these. We also observed phage-like particles in *X. fastidiosa *cells residing in xylem vessels of artificially inoculated almond trees (data not shown).

To further verify the presence of phage particles, we cultured *X. fastidoisa *strain Temecula-1 in 500 ml PW broth for 30 days under the same culture condition as above. A total of 11 batches of cultures were made. Bacterial cells were removed by centrifugation at 5,000 g for 45 minutes. Supernatants of the bacterial cultures were centrifuged once or twice at 12,000 g. The supernatants were then concentrated by high speed centrifugation 155,000 g for 1.5–2 hours. The high speed centrifugation pellets were resuspended in 200–500 μl sterile distilled water and further purified through equilibrium CsCl density gradients. The CsCl density gradients were made up in SM buffer [14]. Briefly, 3-step gradients were 3.4–3.7 ml each of 1.45, 1.5 and 1.7 gm CsCl/ml SM buffer. After layering the resuspended high speed centrifugation pellets (0.2–1.0 ml) on the tops, the gradients were centrifuged at 155,000 g for 18–21 hours and a presumptive phage particle-containing band was observed (data not shown). After removal of samples from the centrifuged gradients, the CsCl was removed by extensive dialysis in SM buffer using Slide-A-Lyzer Mini Dialysis Cassettes per the supplier's (Pierce Biotechnology, Rockland, IL) instructions.

Five μl of phage suspension was added to a 400-mesh copper grid and the droplet was partially wicked off using a triangle-shaped piece of 3 M filter paper. The remaining thin layer of liquid was left on the grid after 3 min. Five μl of 2% uranyl acetate was added to the grid and the droplet partially wicked off after 45 seconds. This procedure was repeated with 5 μl distilled H_2_O, and, after immediate partial wicking of the water droplet, the grid was air-dried. The grids were examined by TEM as described above.

Samples collected from CsCl density gradients revealed the presence of mostly non-tailed icosahedral particles, which could be grouped into two types. The large type particles were about 45 nm (Fig. 1B), similar to those observed from cell pellets (Fig. 1A). No distinct short tails were observed. "Ridges" were sometimes seen on the particle surface. As a control, we used the same negative staining procedure to prepare bacteriophage CP2 from *Xanthomonas citri *subsp. *citri*, a member of the phage family *Podoviridae *[15]. Short tails were readily recognized in CP2 (Fig. 1C). Particles showing a "ridge" were also observed on these particles, suggesting some structural or morphological similarity between CP2 and the *X. fastidosa *particles. The small type icosahedral particles were 30.1 ± 5.0 nm (n = 20) across (Fig. 1D). Interestingly, some of these particles formed an ordered chain (Fig. 1D). Although uncommonly reported, icosahedral phages in ordered chains were observed in ruminal fluid samples of animals [16]. An observed tailed particle is shown in Fig. 1E. The head size was similar to those of the large type of icosahedral particles and the tail was 140 nm long. In addition, we also observed filamentous particles with a width of 17.2 ± 0.5 nm (n = 10) but highly variable in length from 120 to 6,300 nm (Fig. 1F). We are aware that *X. fastidosa *does not posses flagella [1] but type IV pili was reported [17]. However, available information indicated that the width of type IV pili is 5–7 nm [18].

In terms of phage morphology, Ackermann [12] summarized all of the known phages into four morphological groups: tailed, polyhedral, filamentous, and pleomorphic, and 20 Families when nucleic acid and other properties were considered. We observed phage-like particles in the tailed, polyhedral, and pleomorhpic morphological groups. However, the low titer of phages under our experimental conditions and the possible contamination of bacterial chromosomal DNA limited our ability to perform further nucleic acid analyses. Enrichment of phage particles from this fastidious bacterium has been highly challenging. Therefore, we are not able to characterize these particles according to the phage family scheme. However, based on morphology, the large icosahedral particles could belong to the *Podoviridae *but further proof of the presence of short tails is needed; the small icosahedral particles could be in the *Microviridae*; the tailed particles could be in the *Siphoviridae*; and the filamentous particles could be in the *Inoviridae*. Interestingly, all of the four phage families were predicted to be present in *X. fastidiosa *based on prophage sequence analyses [2-6].

We note that the *X. fastidiosa *phages reported here were from late stationary or senescent cultures. This was based on the assumption that prolonged growth in culture would create physical and/or chemical stress to facilitate induction of lysogenic phages into a lytic cycle so that phage particles became visible. We cannot exclude the possibility that some phage particles observed might have been damaged during the preparation process. This could be an explanation of the observed "ridge" formation and the length variation of filamentous particles. Optimization of the phage isolation and purification procedure is needed for future research.

## Conclusion

The presence of different types of phage-like particles resembling those in several bacteriophage families provides new physical evidence, in addition to *X. fastidiosa *genomic information, that *X. fastidiosa *possesses active phages. This is the first report of phage particles released in *X. fastidiosa *cultures.

## Competing interests

The authors declare that they have no competing interests.

## Authors' contributions

JC planned and performed the experiments and prepared the manuscript, EC participated planning the experiments and electron microscopy, and interpreted the data.
